# Sensitive and visual identification of *Chlamydia trachomatis* using multiple cross displacement amplification integrated with a gold nanoparticle-based lateral flow biosensor for point-of-care use

**DOI:** 10.3389/fcimb.2022.949514

**Published:** 2022-07-22

**Authors:** Xu Chen, Wei Yuan, Qingxue Zhou, Yan Tan, Ronghua Wang, Shilei Dong

**Affiliations:** ^1^ The Second Clinical College, Guizhou University of Traditional Chinese Medicine, Guiyang, China; ^2^ Clinical Medical Laboratory of the Second Affiliated Hospital, Guizhou University of Traditional Chinese Medicine, Guiyang, China; ^3^ Quality Control Department, Guizhou Provincial Center for Clinical Laboratory, Guiyang, China; ^4^ Clinical Laboratory, Hangzhou Women’s Hospital, Hangzhou, China; ^5^ Department of Clinical Laboratory, Longli people’s Hospital, Qianlan, China; ^6^ Department of Clinical Laboratory, Zhejiang Hospital, Hangzhou, China

**Keywords:** *Chlamydia trachomatis*, multiple cross displacement amplification, isothermal amplification, gold nanoparticle-based lateral flow biosensor, point-of-care testing

## Abstract

*Chlamydia trachomatis* is the leading cause of bacterial sexually transmitted infection (STI) and remains a major public health challenge, especially in less-developed regions. Establishing a rapid, inexpensive, and easy-to-interpret point-of-care (POC) testing system for *C. trachomatis* could be critical for its treatment and limiting further transmission. Here, we devised a novel approach termed a multiple cross displacement amplification integrated with gold nanoparticle-based lateral flow biosensor (MCDA-AuNPs-LFB) for the highly specific, sensitive, user-friendly, and rapid identification of *C. trachomatis* in clinical samples. A suite of MCDA primers based on the *C. trachomatis ompA* gene from 14 serological variants (serovar A-K, L1, L2, and L3) were successfully designed and used to establish the assay. Optimal assay conditions were identified at 67°C, and the detection procedure, including nucleic acid preparation (approximately 5 min), MCDA amplification (30 min), and AuNPs-LFB visual readout (within 2 min), was completed within 40 min. The all-in cost for each test was approximately $5.5 USD. The limit of detection (LoD) was 10 copies/reaction, and no cross-reaction was observed with non-*C. trachomatis* microbes. A total of 135 suspected *C. trachomatis*-infection genital secretion samples were collected and simultaneously detected using real-time quantitative PCR (qPCR) in our assay. Compared with the qPCR technology, the MCDA-AuNPs-LFB sensitivity, specificity, positive predictive value, and negative predictive value were 100%, 96.20%, 94.92%, and 100%, respectively. Hence, our MCDA-AuNP-LFB assay exhibited considerable potential for POC testing and could be used to identify *C. trachomatis* in clinical settings, particularly in low-income regions.

## Introduction

*Chlamydia trachomatis* (*C. trachomatis*), is an obligate intracellular Gram-negative pathogen and the leading cause of approximately 130 million new bacterial sexually transmitted infections (STIs) each year (World Health Organization, 2016; Woodhall et al., 2018). Chlamydial infection is a major global public health concern, affecting quality of life and causing serious morbidity and mortality, especially in low-income regions, including Africa, Asia, the Middle East, and South America (Adachi et al., 2016; Molano et al., 2018). Asymptomatic infections are common in both female and male patients, but if untreated in a timely manner, infections may cause severe complications, such as infertility, salpingitis, chronic pelvic pain, epididymitis, and orchitis (Poston et al., 2019; Murray and McKay, 2021). Maternal infection is associated with serious adverse pregnancy outcomes, including miscarriage, stillbirth, low birth weight, preterm birth, or direct fetal infection (Adachi et al., 2016; He et al., 2020). Furthermore, *C. trachomatis* is a cofactor in human immunodeficiency virus transmission and human papillomavirus related-cervical cancer (Goulart et al., 2020; Buckner et al., 2016; Naldini et al., 2019). Therefore, establishing a specific, sensitive, rapid, inexpensive, and easy-to-interpret point-of-care (POC) testing system for *C. trachomatis* would be important for treatment and limiting transmission.

In POC nucleic acid testing, multiple cross displacement amplification (MCDA) is a novel isothermal amplification approach and is an attractive alternative to traditional nucleic acid amplification procedures such as PCR and associated methods, multiple PCR, nested PCR, and real-time PCR-MCDA is highly specific, sensitive (from 7 to 20 genome copies), robust, cost-effective, easy-to-operate, and does not require costly thermocycling facilities (Wang et al., 2015; Obande and Banga, 2020; Jiao et al., 2021). The strategy was previously used for the rapid detection of various pathogens, including SARS-CoV-2, *Neisseria gonorrhoeae*, and *Candida tropicalis* (Li et al., 2020; Chen et al., 2021; Wang Y. et al., 2021). In the MCDA system, nucleic acid isothermal amplification is completed using *Bacillus stearothermophilus* (*Bst*) DNA polymerase with strand-displacement activity for amplifying the target gene at a constant temperature (Wang et al., 2015). To specifically amplify target nucleic acid sequences, 10 primers are designed to span 10 distinct target fragment regions, including 2 cross primers (CP1 and CP2), 2 displacement primers (F1 and F2), and 6 amplification primers (C1, C2, D1, D2, R1, and R2) (Wang et al., 2015; Wang et al., 2016).

The gold nanoparticle-based lateral flow biosensor (AuNPs-LFB) is a paper-based platform and highly attractive for POC diagnostics; it is easy to manufacture, inexpensive, sensitive, and specific, and robustly and rapidly detects targets (Aldewachi et al., 2017; Huang et al., 2019; Wang T. et al., 2021). AuNPs are the most common nanomaterials used as optical labels in LFB; they are easily synthesized, biocompatible, size-tunable, stable over time, and display a strong red signal visible to the naked eye (Quesada-González and Merkoci, 2015; Cui and Zhou, 2020). Based on these properties, AuNPs-LFB have wide applications, including infectious disease, food, and water safety monitoring, and many other medical uses (Banerjee and Jaiswal, 2018; Kim et al., 2019).

**Figure 1:**
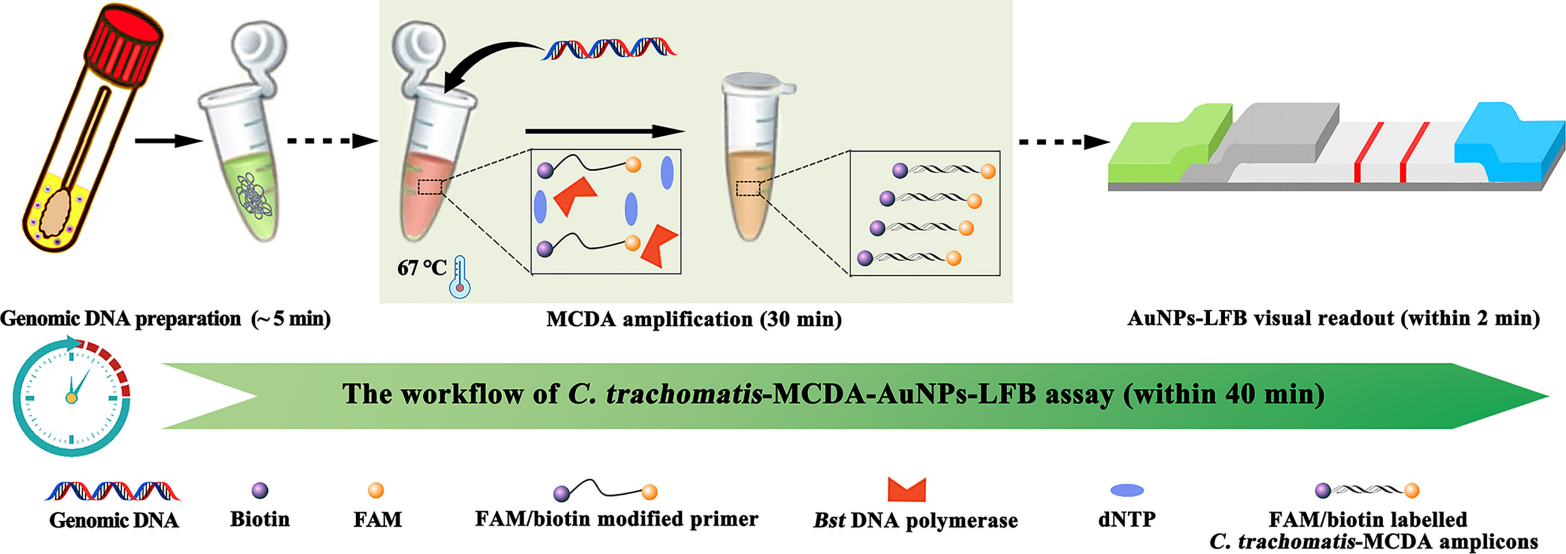
C*. trachomatis*-MCDA-AuNPs-LFB assay workflow. The workflow includes genomic DNA preparation, MCDA amplification, and AuNP-LFB visual interpretation, all completed within 40 min.

**Figure 2:**
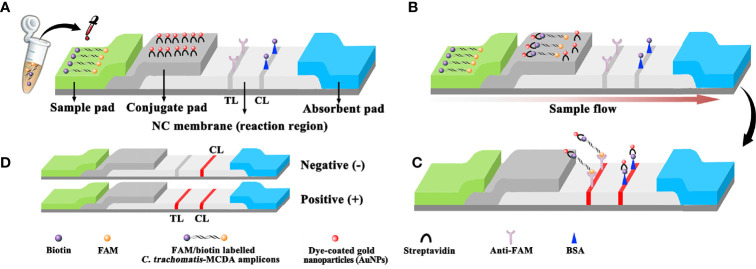
Schematic diagram showing AuNPs-LFB principles for the visual identification of *C trachomatis*-MCDA amplification products. **(A)** *C trachomatis*-MCDA amplification products (0.5 μl) and running buffer (100 μl) were simultaneously added to the sample pad. **(B)** Due to capillary action, the running buffer, containing **(C)** *trachomatis*-MCDA products, moved forward onto the conjugate pad and nitrocellulose (NC) membrane. Streptavidin-AuNPs were hydrated, rapidly released, and combined with *C trachomatis*-MCDA products at the conjugate pad. **(C)** FAM/biotin-labeled *C trachomatis*-MCDA products were arrested by anti-FAM at the TL strip, and streptavidin-DPNs were arrested at the biotin-BSA CL strip. **(D)** Interpretation of the *C trachomatis*-AuNP-LFB assay. For a positive result, both the CL and TL appeared on the biosensor. For a negative result, only the CL was observed on the AuNP-LFB. TL: test line; CL: control line.

In this study, an MCDA-AuNPs-LFB was devised for the visual and rapid detection of *C. trachomatis* by targeting the *ompA* gene from several serovars (A–K, L1, L2, and L3) (Somboonna et al., 2018; Molano et al., 2018). This gene showed no homology with other microbial genomes from BLAST searches in GenBank. *C. trachomatis* MCDA-AuNPs-LFB principles and workflow are shown (**Figures 1**, **2**, respectively). The complete diagnostic process is accomplished within 40 min, and assay feasibility was validated using clinical genital secretion samples from patients. Therefore, the MCDA-AuNPs-LFB assay may be used for POC testing and identifying *C. trachomatis* infectious, especially in resource-limited regions.

## Materials and methods

### Reagents

We purchased AuNP-based LFB materials, including crimson red dye streptavidin-coated AuNPs (SA-AuNPs, 40 ± 5 nm) from Bangs Laboratories Inc. (IN, USA) and biotinylated bovine serum albumin (biotin-BSA) and a rabbit anti-fluorescein antibody (anti-FAM) from Abcam Co., Ltd. (Shanghai, China). Four LFB sections, including nitrocellulose membranes, conjugate, absorption, and absorption pads, were manufactured and laminated on plastic adhesive backing by HuiDeXing Biotech. Co., Ltd. (Tianjing, China) according to our design scheme (**Figure 2**). Nucleic acid releasers were obtained from BaiAoLaiBo Technique Ltd. (Beijing, China). Universal isothermal amplification kits and the colorimetric indicator malachite green (MG) were purchased from HuiDeXin Biotech (Tianjin, China). Commercial PCR diagnostic kits for *C. trachomatis* were purchased from DaAn Gene Co., Ltd. (Guangzhou, China).

### Preparing target DNA and clinical samples

Full-length *ompA* sequences from 14 C*. trachomatis* serological variants (A, B, C, D, E, F, G, H, I, J, K, L1, L2, and L3, with accession numbers JX548318.1, JX559518.1, JX559519.1, KP164991.1, JX559522.1, JX564244.1, JX564245.1, JX564246.1, JX564247.1, JX648604.1, JX564248.1, JX569832.1, KP120855.1, and JX569834.1, respectively) were synthesized and cloned into the pUC57 vector by Tsingke Biotech (Beijing, China). The initial concentration of each plasmid was 1 × 10^8^ copies. The *C. trachomatis* serovar A plasmid was used as a positive control.

We collected 135 suspected *C. trachomatis*-infected genital secretion samples from patients at Hangzhou Women’s Hospital between July 2021 and March 2022. Crude genomic DNA was extracted using Nucleic Acid Releasing Agents (BaiAoLaiBo Technique Ltd. Beijing, China) (Cat. # BTN61202) in accordance with the manufacturer’s instructions. Briefly, a genital secretion sample was mixed with 100 μl of Nucleic Acid Releasing Agent for 5 min cell lysis, and the supernatant was used as a template for *C. trachomatis*-MCDA assay. The *Bst* DNA polymerase is less affected for inhibitors in an MCDA reaction compared to *Thermus aquaticus* DNA polymerase in conventional PCR (Somboonna et al., 2018). Genomic DNA concentrations were measured using a Nano-Drop ND-2000 instrument (Thermo, USA) at A260/280. Other microorganisms used in this study are shown (**Table 1**).

**Table 1:** Microbial strains used in this study.

No.	Pathogen	Source of pathogens^a^	No. of strains	*C. trachomatis*-MCDA-AuNPs-LFB result^b^
1 2 3 4 5 6 7 8 9 10 11 12 13 14 15 16 17 18 19 19 20 21 22 23 24 25 26 27 28 29 30 31 32	*C. trachomatis* serovar A *ompA*-plasmids *C. trachomatis* serovar B *ompA*-plasmids *C. trachomatis* serovar C *ompA*-plasmids *C. trachomatis* serovar D *ompA*-plasmids *C. trachomatis* serovar E *ompA*-plasmids *C. trachomatis* serovar F *ompA*-plasmids *C. trachomatis* serovar G *ompA*-plasmids *C. trachomatis* serovar H *ompA*-plasmids *C. trachomatis* serovar I *ompA*-plasmids *C. trachomatis* serovar J *ompA*-plasmids *C. trachomatis* serovar K *ompA*-plasmids *C. trachomatis* serovar L1 *ompA*-plasmids *C. trachomatis* serovar L2 *ompA*-plasmids *C. trachomatis* serovar L3 *ompA*-plasmids *C. trachomatis* (clinical samples) *Ureaplasma urealyticum* *Neisseria gonorrhoeae* *Escherichia coli* *Staphylococcus aureus* Human papilloma virus Human rhinovirus Coxsackie virus CAV16 Human enterovirus EV71 *Mycoplasma pneumoniae* *Listeria monocytogenes* *Haemophilus influenza* *Cryptococcus neoformans* *Bordetella pertussis* *Streptococcus pyogenes* *Candida glabrata* *Pseudomonas aeruginosa* *Shigella flexneri* *Klebsiella pneumoniae*	Constructed by Tsingke Biotech (Beijing, China) Constructed by Tsingke Biotech (Beijing, China) Constructed by Tsingke Biotech (Beijing, China) Constructed by Tsingke Biotech (Beijing, China) Constructed by Tsingke Biotech (Beijing, China) Constructed by Tsingke Biotech (Beijing, China) Constructed by Tsingke Biotech (Beijing, China) Constructed by Tsingke Biotech (Beijing, China) Constructed by Tsingke Biotech (Beijing, China) Constructed by Tsingke Biotech (Beijing, China) Constructed by Tsingke Biotech (Beijing, China) Constructed by Tsingke Biotech (Beijing, China) Constructed by Tsingke Biotech (Beijing, China) Constructed by Tsingke Biotech (Beijing, China) Hangzhou Women’s Hospital Hangzhou Women’s Hospital Hangzhou Women’s Hospital 2nd GZUTCM 2nd GZUTCM GZCCL GZCCL GZCCL GZCCL Zhejiang Hospital Zhejiang Hospital ATCC49247 ATCC13690 GZCCL 2nd GZUTCM 2nd GZUTCM 2nd GZUTCM Zhejiang Hospital Zhejiang Hospital	1 1 1 1 1 1 1 1 1 1 1 1 1 1 7 1 1 1 1 1 1 1 1 1 1 1 1 1 1 1 1 1 1	P P P P P P P P P P P P P P P N N N N N N N N N N N N N N N N N N

a ATCC, American Type Culture Collection; 2nd GZUTCM, the Second Affiliated Hospital, Guizhou University of Traditional Chinese Medicine; GZCCL, Guizhou Provincial Center for Clinical Laboratory.

b P, Positive; N, Negative.

### Preparing AuNPs-LFB

The AuNPs-LFB (60 mm × 4 mm) is shown (**Figure 2**). Briefly, the LFB was constructed from four sections, including nitrocellulose membranes, sample, conjugate, and absorption pads. All were laminated on a plastic adhesive backing card. Crimson red dye SA-AuNPs were deposited onto the conjugate pad. Biotin-BSA (4 mg·ml^−1^) and anti-FAM (0.2 mg·ml^−1^) were immobilized onto the nitrocellulose membrane to function as a control line (CL) and test line (TL), respectively, with 5-mm separating lines. For AuNPs-LFB detection, 0.5 μl of MCDA products and 100 μl of running buffer (100 mM PBS, 1% Tween 20, pH 7.4) were simultaneously dropped onto the sample pad, and the solution flowed along the LFB *via* capillary action. Finally, results were generated on the nitrocellulose membrane (crimson red line) within 2 min.

### MCDA primer design

A suite of 10 MCDA primers was designed to amplify 10 different sections of *C. trachomatis ompA*. *ompA* genes from 14 C*. trachomatis* serological variants (serovar A, B, C, D, E, F, G, H, I, J, K, L1, L2, and L3) were aligned using MEGA-X software (https://www.megasoftware.net/) (**Supplementary Figure S1**) and conserved sequences selected for MCDA primer design. MCDA degenerate primers, including a pair of cross primers (CP1 and CP2), a pair of displacement primers (F1 and F2), and three pairs of amplification primers (C1, C2, D1, D2, R1, and R2) were generated using Primer Explorer V5 (http://primerexplorer.jp/e/) and PRIMER PREMIER 5.0 software. Primer specificity was verified by the BLAST analysis tool. *C. trachomatis*-MCDA primer sequences and modifications are shown (**Table 2**). Primers were synthesized and purified by Tsingke Biotech using a high-performance liquid chromatography method.

**Table 2:** **C** *. trachomatis*-MCDA-AuNPs-LFB degenerate primers used in this study.

Primer name	Sequence and modifications	Length	Gene
F1	5′-CCGCTTTGAGTTCTGCT-3′	17 nt	*ompA*
F2	5′-CCAT(C/T)T(G/C)(A/G)AA(C/T)TCTTTATTCACATC-3′	25 nt	
CP1	5′-(C/T)AGAATTCCGTCGATCATAAGGCTTCCTCCTTGCAAGCTCTG-3′	42 mer	
CP2 C1* C2	5′-GC(A/G)CCACTTGGTGTGACG(A/T)GTTT(G/T)CAAAAC(A/G)CGGTCG-3′ 5′-Biotin-(C/T)AGAATTCCGTCGATCATAAGGCTT-3′ 5′-GC(A/G)CCACTTGGTGTGACG-3′	37 mer 25 nt 18 nt	
D1* D2 R1 R2	5′-FAM-TCAGCAGGATTCCCCAC-3′ 5′-CATGCG(C/T)(A/G)T(G/T)GGTTACT-3′ 5′-CC(A/G)CC(A/G)AAACCTTCCCA-3′ 5′-AGATCCTTGCGATCCTT-3′	17 nt 17 nt 17 nt 17 nt	

C1*, 5′-labeled with biotin when used for the AuNP-LFB assay; D1*, 5′-labeled with FAM when used for the AuNP-LFB assay.

FAM, 6-carboxy-fluorescein; nt, nucleotide; mer, monomeric unit.

### MCDA reactions and detection

MCDA amplification was conducted in a one-step 25-μl reaction volume as previously described (Li et al., 2020): 1 μl of standard plasmid template (5 μl of clinical sample template); 1.6 μM each CP1 and CP2; 0.4 μM each F1 and F2; 0.8 μM each C1*, C2, D1*, D2, R1, and R2; 1 μl of *Bst* 2.0 DNA polymerase (8 U); 1 μl of AMV reverse transcriptase (10 U) (only for RNA templates); 1 μl of colorimetric indicator (MG); 12.5 μl of 2× reaction buffer (2 M betaine, 16 mM MgSO_4_, 40 mM KCl, 20 mM (NH_4_)_2_SO_4_, 40 mM Tris-HCl (pH 8.8), and 0.2% Tween-20); double-distilled water (DW) was added to 25 μl. The reaction process was performed in a heat block or water bath at a fixed temperature (optimization conditions are outlined below).

Amplification products were examined using real-time turbidity LA-500 (Lumiprobe, Japan), visual detection reagents (MG), and AuNPs-LFB methods. A real-time turbidity value of >0.1 indicated a positive outcome. For visual MG analysis, reaction mixtures changed to light green, suggesting a positive outcome, while colorless mixtures indicate negative results. For AuNPs-LFB detection, both CL and TL simultaneously appeared on the biosensor and demonstrated a positive result, but for negative outcomes, only the CL appeared.

### Optimizing assay conditions

To determine optimal *C. trachomatis*-MCDA-AuNPs-LFB reaction conditions (reaction temperatures and incubation times), MCDA reactions were incubated at 63°C–70°C (at 1°C intervals) and amplicons were analyzed by real-time turbidity. Incubation times, ranging from 20 min to 50 min (at 10-min intervals) were tested under the optimal reaction temperature. Results were simultaneously monitored using MG and AuNPs-LFB. Each assay was conducted in triplicate.

### Assay sensitivity and specificity

To determine the limit of detection (LoD) of the assay system, *ompA* standard plasmid templates were 10-fold serially diluted: 5.0 × 10^4^–5.0 × 10^−1^ copies. Assays were performed under optimal reaction conditions, and results were interpreted using MG and AuNP-LFB. Assay specificity was tested by comparing *C. trachomatis* DNA templates (serovar A–K, L1, L2, and L3) with nucleic acids from other pathogens (at least 1.0 × 10^4^copies/test). DW was used as a blank control and assays were performed at least three times.

### Assay verification and feasibility using clinical samples

To verify assay feasibility, optimally derived assays were tested using clinical genital secretion specimens. Using 135 suspected *C. trachomatis*-infected genital secretion samples from Hangzhou Women’s Hospital (Hangzhou, China), we compared our assay with a commercial *C. trachomatis* real-time TaqMan PCR method (DaAn Gene Co., Ltd. China) (Cat. #DA0071) on an Applied Biosystems™ 7500 Real-Time PCR System (Life Technologies, Singapore), which was used as a reference method since it is commonly used in clinical Chinese laboratories, and its sensitivity was verified using *C. trachomatis* standard substance (Guangzhou BDS Biological Technology Co., Ltd.). According to the manufacturer’s instructions, *C. trachomatis* concentrations > 500 copies indicated a positive outcome. All detection studies were conducted at biosafety level 2 according to the WHO laboratory biosafety manual, 3rd edition. Our assay data were compared with *C. trachomatis* real-time TaqMan PCR detection data. The statistical parameters were calculated using the online tool from MedCalc (http://www.medcalc.org/calc/diagnostic_test.php) (Jevtuševskaja et al., 2016).

## Results

### Assay system overview

The *C. trachomatis*-MCDA-AuNPs-LFB assay mechanism and workflow are shown (**Figures 1**, **2**). Briefly, *C. trachomatis* DNA templates were generated by nucleic acid agents. Two core primers, C1 and D1, were modified at 5′-ends with biotin and FAM, respectively. Target DNA was pre-amplified using MCDA reactions at 67°C for 30 min and amplicons were simultaneously labeled with biotin and FAM. Finally, the MCDA products were monitored visually through AuNPs-LFB within 2 min.

After *C. trachomatis*-MCDA reactions, 0.5 μl of MCDA products and 100 µl of running buffer (100 mM PBS, 1% Tween 20, pH 7.4) were simultaneously added to the biosensor sample pad (**Figure 2A**). The running buffer that contained MCDA products moved along the biosensor *via* capillary action and rehydrated immobilized crimson red dye SA-AuNPs in the conjugate pad (**Figure 2B**). For positive results, FAM/biotin-labeled *ompA*-MCDA amplicons were specifically captured by anti-FAM at the TL, and SA-AuNPs were captured by biotin-BSA at the CL. For negative result, only SA-AuNPs were captured by biotin-BSA at the CL (**Figure 2C**). Interpretation of the *C. trachomatis*-MCDA-AuNP-LFB assay is outlined (**Figure 2D**). The AuNPs-LFB was also user-friendly and inexpensive (~$2.0 USD/test). Therefore, the all-in cost for each test, including nucleic acid preparation (~$0.5 USD), MCDA reactions (~$3.0 USD), and AuNP-LFB detection (~ $2.0 USD) was approximately $5.5 USD.

### Confirming the *C. trachomatis* MCDA assay

**Figure 3:**
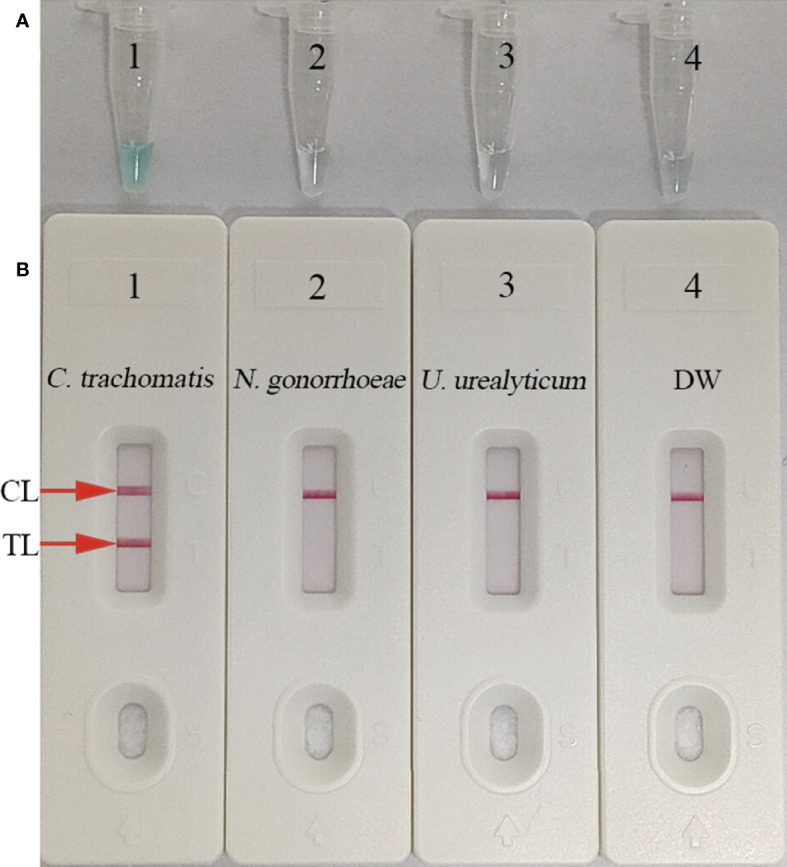
Confirmation and verification of **(C)** *trachomatis*-MCDA products. *C trachomatis*-MCDA products were measured simultaneously using malachite green (MG) **(A)** and AuNPs-LFB **(B)**. Tube 1/Biosensor 1: positive result for *C trachomatis ompA* standard plasmids; Tube 2/Biosensor 2: negative result for *Neisseria gonorrhoeae*; Tube 3/Biosensor 3: negative result for *Ureaplasma urealyticum*; Tube 4/Biosensor 4: blank control (distilled water, DW). TL: test line; CL: control line.

To validate the reaction system, MCDA amplification mixtures were incubated at a fixed temperature of 65°C for 1 h, while *C. trachomatis ompA* standard plasmids were used as templates. Results were simultaneously indicated by MG and AuNP-LFB methods. *C. trachomatis*-MCDA mixtures changed to light green, while *N. gonorrhoeae*, *Ureaplasma urealyticum*, and the blank control (DW) remained colorless (**Figure 3A**). Both CL and TL appeared in *C. trachomatis*-MCDA amplification mixtures, while only CL was observed in negative and blank controls (**Figure 3B**). Thus, the suite of MCDA primers for *C. trachomatis*-MCDA reaction was validated.

### Determining an optimal reaction assay temperature

**Figure 4:**
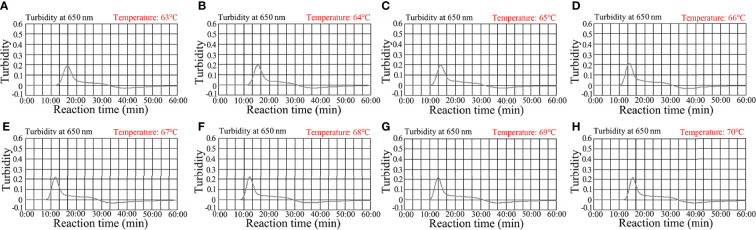
Optimizing the temperature for the *C. trachomatis*-MCDA assay. *C. trachomatis*-MCDA amplification of *ompA* was monitored using real-time turbidity. Corresponding amplicon concentration curves are marked in graphs. Turbidity > 0.1 indicated a positive value. **(A–H)** Eight kinetic graphs were generated at different temperatures (63°C–70°C at 1°C intervals) with *C. trachomatis ompA*-plasmids at 1 × 10^3^ copies. Graph E (67°C) showed the fastest and most robust amplification.

To determine an optimal amplification temperature, we tested a 60°C–70°C temperature range with 1.0 × 10^3^ *C. trachomatis ompA*-plasmid copies (**Figures 4A–H**. Real-time turbidity indicated that faster and robust *C. trachomatis*-MCDA amplification occurred at 67°C (**Figure 4E**). Thus, 67°C was the most suitable amplification temperature for assay conditions.

### Assay sensitivity

**Figure 5:**
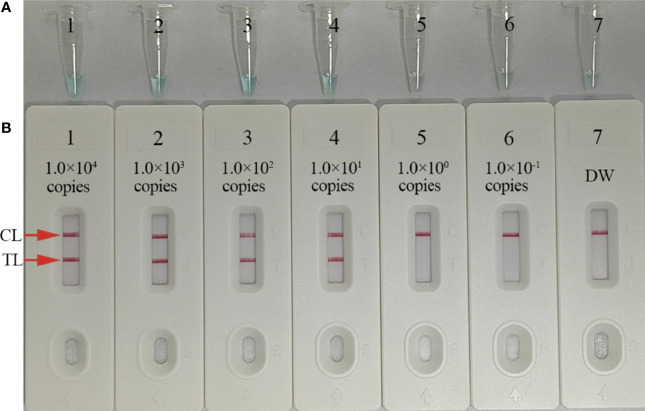
Sensitivity analysis of *C trachomatis*-MCDA-AuNPs-LFB using *C trachomatis ompA*-plasmid serial dilutions. Serial dilutions (1.0 × 10^4^, 1.0 × 10^3^, 1.0 × 10^2^, 1.0 × 10^1^, 1.0 × 10^0^, and 1.0 × 10^−1^ copies) of *C trachomatis ompA*-plasmids were used as templates, and distilled water (DW) was used as the negative control. Results were simultaneously analyzed by malachite green (MG) **(A)** and AuNPs-LFB **(B)**. The limit of detection (LoD) for *C trachomatis*-MCDA-AuNP-LFB was 10 copies/test. CL, control line; TL, test line.

To test the LoD of our assay, we prepared 10-fold serial dilutions (1.0 × 10^4^–1.0 × 10^−1^ copies/test) of *C. trachomatis ompA* standard plasmids. MCDA reactions were conducted as described and results were visualized by MG and AuNPs-LFB. These studies suggested that *C. trachomatis* DNA templates were sufficiently detected at 10 copies/test (**Figures 5A, B**).

### Optimizing the assay reaction time

**Figure 6:**
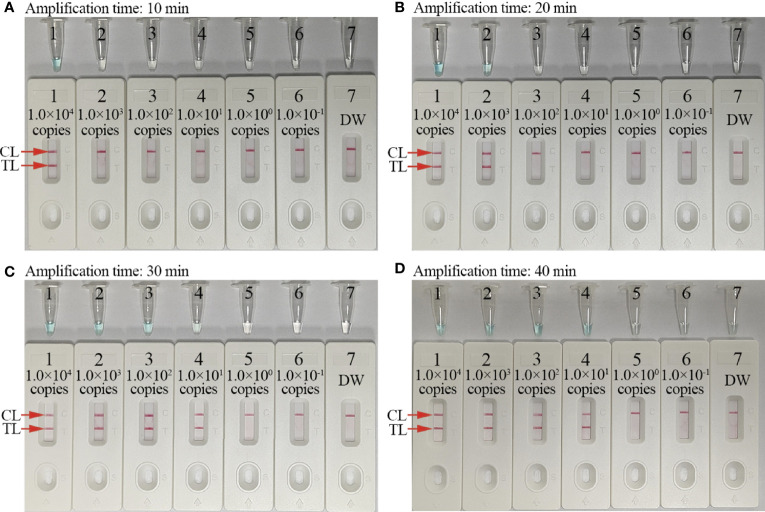
Optimal amplification time for the *C. trachomatis*-MCDA-AuNPs-LFB assay. Four reaction times (**A**, 10 min; **B**, 20 min; **C**, 30 min; and **D**, 40 min) were evaluated at 67°C. Tubes/biosensors 1–7 represented *C. trachomatis ompA* template levels: 1.0 × 10^4^, 1.0 × 10^3^, 1.0 × 10^2^, 1.0 × 10^1^, 1.0 × 10^0^, 1.0 × 10^−1^ copies, and negative control (distilled water, DW), respectively. Results were simultaneously analyzed using malachite green (MG) and AuNP-LFB. The optimal limit of detection (LoD) occurred when the amplification lasted for 30 min **(C)**. CL: control line; TL: test line.

To optimize assay reaction times during isothermal amplification stages, different reaction times (20, 30, 40, and 50 min) were tested at 67°C. *C. trachomatis*-MCDA products were visualized by MG and AuNPs-LFB. These results confirmed that the LoD of the *C. trachomatis* DNA template (10 copies/reaction) was tested when the MCDA reaction sustained 30 min (**Figure 6**). Hence, 30 min was chosen as an optimal reaction time for amplification.

### Assay specificity

**Figure 7:**
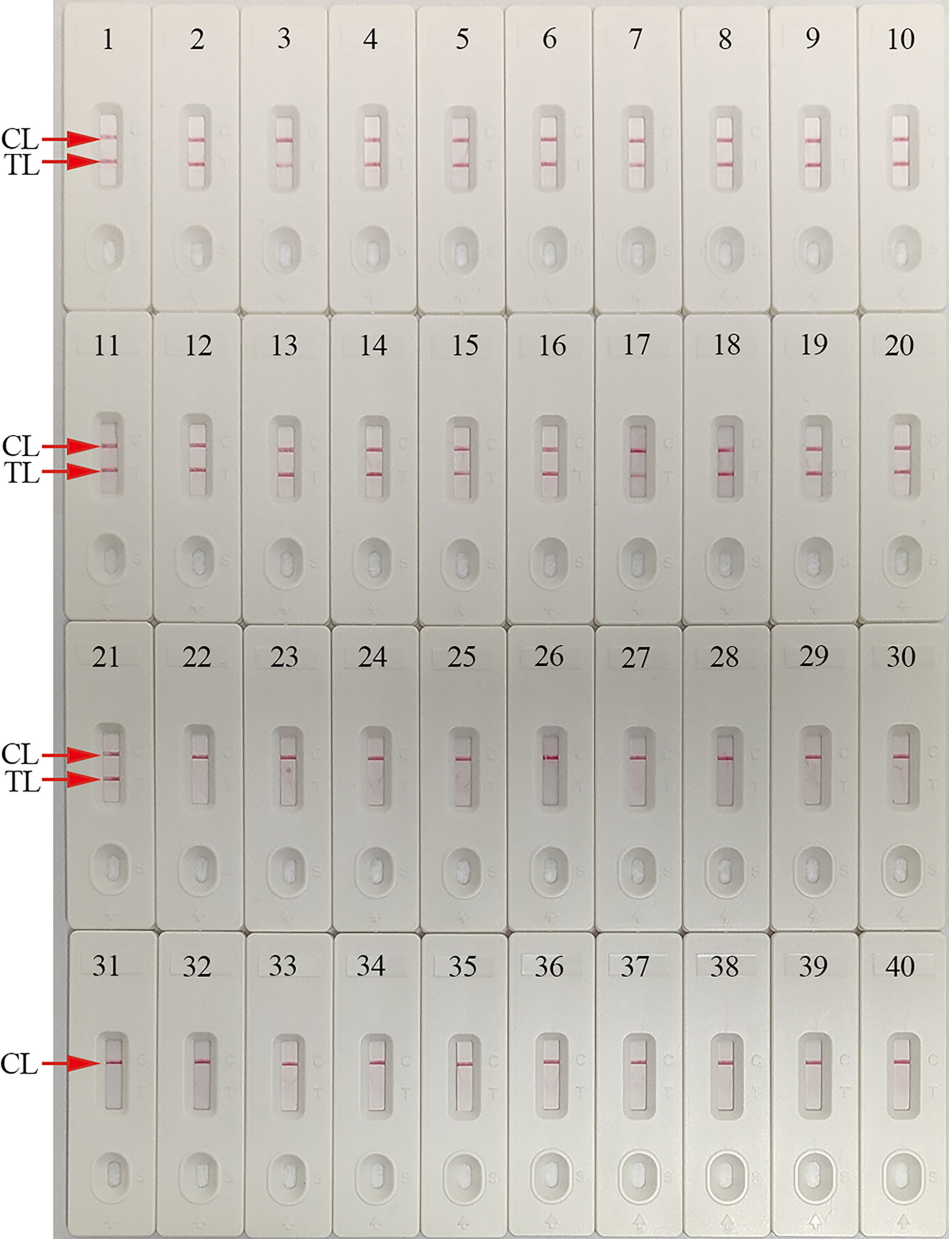
Analytical specificity of the *C. trachomatis*-MCDA-AuNPs-LFB assay using different strains. Assay specificity was evaluated using different nucleic acids as temperatures, and products were tested using AuNPs-LFB. Biosensors 1–14, *C. trachomatis* serovars A, B, C, D, E, F, G, H, I, J, K, L1, L2, and L3 *ompA*-plasmids; Biosensors 15–21, *C. trachomatis* (clinical samples); Biosensor 22, *Ureaplasma urealyticum*; Biosensor 23, *Neisseria gonorrhoeae*; Biosensor 24, *Escherichia coli*; Biosensor 25, *Staphylococcus aureus*; Biosensor 26, Human papilloma virus; Biosensor 27, Human rhinovirus; Biosensor 28, Coxsackie virus CAV16; Biosensor 29, Human enterovirus EV71; Biosensor 30, *Mycoplasma pneumoniae*; Biosensor 31, *Listeria monocytogenes*; Biosensor 32, *Haemophilus influenza*; Biosensor 33, *Cryptococcus neoformans*; Biosensor 34, *Bordetella pertussis*; Biosensor 35, *Streptococcus pyogenes*; Biosensor 36, *Candida glabrata*; Biosensor 37, *Pseudomonas aeruginosa*; Biosensor 38, *Shigella flexneri*; Biosensor 39, *Klebsiella pneumoniae*; Biosensor 40, negative control (distilled water, DW). CL: control line; TL: test line.

This parameter was determined using *C. trachomatis omp A*-plasmids (serovar A, B, C, D, E, F, G, H, I, G, K, L1, L2, and L3), *C. trachomatis*-positive clinical samples (confirmed by qPCR), and non-*C. trachomatis* strains. Only *C. trachomatis* strains generated positive results, while positive results were absent for non-*C. trachomatis* strains (**Figure 7**). Hence, our detection system was highly specific for identifying *C. trachomatis*.

### Assay evaluation using clinical samples

To evaluate assay feasibility and accuracy, 135 suspected *C. trachomatis*-infection genital secretion samples were simultaneously detected using qPCR and our assay; 56 (41.5%) were positive with the qPCR method (> 500 copies), while all *C. trachomatis*-positive samples were verified using our assay. Also, 3/79 “negative samples” (ranging from 100 to 500 copies) were positive using our assay (**Table 3** and **Supplementary Table S1**). In order to verify the three discordant results, the three samples were amplified with PCR, and the amplicons were tested using DNA sequencing. The results were consistent with MCDA-AuNPs-LFB and presented positive outcomes (**Supplementary Table S1**). Compared with the qPCR technology (*C. trachomatis* real-time TaqMan PCR method, DaAn Gene Co., Ltd. China), the MCDA-AuNPs-LFB sensitivity, specificity, positive predictive value, and negative predictive value were 100%, 96.20%, 94.92%, and 100%, respectively (**Table 2**). Thus, our assay demonstrated a higher sensitivity and specificity for identifying *C. trachomatis*-infected patients, especially those with low bacterial loads at initial infection stages.

**Table 3:** Comparing *C. trachomatis* levels in clinical samples using our MCDA-AuNPs-LFB assay with a qPCR method.

*C. trachomatis*-MCDA-AuNPs-LFB	*C. trachomatis* real-time TaqMan PCR(reference method)			Sensitivity (%)	Specificity (%)	PPV^a^ (%)	NPV^b^ (%)
	Positive	Negative	Total				
Positive	56	3	59	100	96.20	94.92	100
Negative	0	76	76				
Total	56	79	135				

a PPV, positive predictive value;

b NPV, negative predictive value.

## Discussion

In this study, we successfully established an accurate, rapid, easy-to-interpret, inexpensive, specific, and sensitive POC *C. trachomatis* testing system, using MCDA for *ompA* amplification, followed by an AuNPs-LFB visual specific readout. Our assay was robustly assessed using suspected *C. trachomatis*-infection genital secretion samples and compared with a commercial *C. trachomatis* real-time TaqMan PCR kit (DaAn Gene Co., Ltd. China). Nucleic acid amplification tests (NAATs), such as qPCR, nested PCR, and reverse transcription PCR, are the mainstay methods for identifying infections due to their high specificity and sensitivity (Phillips et al., 2018; Xu et al., 2022). However, some NAATs are unaffordable and inaccessible in less-developed regions as they require robust laboratory infrastructures, expensive instruments, and trained personnel (Kelly et al., 2017; Adamson et al., 2020). Here, our POC system required simple devices: a heating block or water bath that maintained 67°C for 30 min to perform pre-amplification steps. Also, results can be read out using an AuNPs-LFB method. Hence, our assay procedure, including nucleic acid extraction (approximately 5 min), MCDA (30 min), and visual results readout (approximately 2 min), was completed within 40 min.

Isothermal amplification technologies, including loop-mediated isothermal amplification (LAMP) and cross-priming amplification (CPA), have also been used to identify *C. trachomatis*. Somboonna et al. used LAMP assays for *C. trachomatis* and identified a LoD of 11.25 target DNA copies within 1.0 h (Somboonna et al., 2018). Yu et al. used CPA and identified 45 target copies in <1.5 h (Yu et al., 2016). MCDA is as a novel isothermal amplification approach, first devised by Wang et al. (2015) and is more sensitive than traditional PCR and other isothermal amplification methods. In the MCDA system, a suite of 10 specific primers with 10 binding sites for the target gene provide high specificity (Jiang et al., 2021; Luu et al., 2021). In our study, MCDA primers based on *C. trachomatis ompA* were successfully designed. Assay specificity was verified using several *C. trachomatis* serological variants (A–K, L1, L2, and L3) and other pathogens, such that our assay specifically identified *C. trachomatis* strain, while no cross-reactions were identified with non-*C. trachomatis* pathogens (**Table 3** and **Figure 7**). Additionally, our assay detected the target gene as low as 10 copies/reaction, which was more sensitive than LAMP and CPA assays. Assay feasibility and accuracy were also verified using clinical genital secretion samples. More importantly, 3/79 “negative samples” (*C. trachomatis* concentrations < 500 copies) were positive by our assay (**Table 2** and **Supplementary Table S1**). Thus, our assay demonstrated higher sensitivity than the *C. trachomatis* real-time TaqMan PCR method (DaAn Gene Co., Ltd. China).

To rapidly and visually analyze *C. trachomatis*-MCDA amplification products, the AuNPs-LFB was used. AuNPs-LFB is a paper-based assay platform and is extremely applicable to POC testing as it is robust, inexpensive, user-friendly, sensitive, and specific (Koczula and Gallotta, 2016; Ye et al., 2020; Wang T. et al., 2021). Importantly, these parameters fulfilled ASSURED POC testing criteria (affordable, sensitive, specific, user-friendly, robust, equipment-free, and deliverable) as recommended by WHO (Wang Y. et al., 2021). Our AuNPs-LFB contained four components: a nitrocellulose membrane, sample, conjugate, and adsorbent pads. The sample pad was composed of cellulose and was ideal for transporting *C. trachomatis*-MCDA products to the next biosensor component. Crimson red dye SA-AuNPs were loaded onto the conjugate pad, while anti-FAM and BSA-biotin were fixed to the TL and CL of the nitrocellulose membrane, respectively. For positive *C. trachomatis*-MCDA products, FAM/biotin-labeled MCDA amplicons were captured by anti-FAM at the TL, while SA-AuNPs were arrested by biotin-BSA at the CL. For negative outcomes, only SA-AuNPs were integrated with biotin-BSA at the CL. The adsorbent pad is placed at the end of the biosensor and serves as bibulous paper to prompt the *C. trachomatis*-MCDA product flow from the sample pad to the nitrocellulose membrane. Real-time turbidity and MG reagents were also used to analyze *C. trachomatis*-MCDA products; the former method required specific instrumentation while the latter was ambiguous when MCDA product concentrations were low (**Figure 6C**).

Our study also had some drawbacks. Firstly, for further evaluation of our assay, it needs to be compared with a highly sensitive method as reference, including more samples with low copy numbers. Secondly, our assay can be used for qualitative detection of *C. trachomatis*, but not for measurement of the concentrations of *C. trachomatis* in sample; the quantitative determination of MCDA-AuNPs-LFB could be further studied in the future. Thirdly, *C. trachomatis*-MCDA reaction tubes must be taken off for AuNP-LFB detection. Thus, there is a risk of carry-over contamination. To limit this, spraying timely 10%–15% sodium hypochlorite solution and 70% ethanol after completion of each AuNP-LFB assay is an effective measure to avoid nucleic acid contamination in laboratory. In our study, we observed no false-positive outcomes in non-*C. trachomatis* strains; thus, cross-contamination was effectively controlled.

## Conclusions

We integrated MCDA isothermal amplification with a visual AuNPs-LFB readout to devise a novel approach for the rapid, highly specific, sensitive, user-friendly, and visual identification of *C. trachomatis* in clinical settings. Termed *C. trachomatis*-MCDA-AuNPs-LFB, the LoD was 10 copies/reaction, and importantly, the assay showed no cross-reactions with non-*C. trachomatis* microbes. The detection procedure was completed within 40 min and did not require expensive instrumentation. Hence, our novel assay has great potential for the POC testing and identification of *C. trachomatis* in clinical settings, particularly in low-income regions.

## Data availability statement

The original contributions to the study are included in the article/**Supplementary Material**. Further enquiries can also be directed to the corresponding author.

## Ethics statement

This study was approved by the Human Ethics Committee of Hangzhou Women’s Hospital (Approval No. [2021]-K (2)-8) and complied with the Declaration of Helsinki. Before our team obtained clinical samples/isolates and conducted this research, any personal patient identifiers were removed. Patient informed consent was waived by the ethics committee.

## Author contributions

XC was involved in study conceptualization, project administration, supervision, investigation, validation, experiments, original draft preparation, and funding acquisition. WY and QZ collected clinical samples and performed experiments. YT curated data and performed project administration, while RW collected clinical samples and performed validation studies. XC and WY curated data and performed experiments. SD was involved in conceptualization, project administration, supervision, validation, funding acquisition, and reviewing and editing. All authors contributed to the article and approved the submitted version.

## Funding

This work was supported by the Program of Scientific and Technological Project in Guizhou Province (Grant No. Qian Ke He [2020]4Y184), the Program of Science and Technology of Guizhou Provincial Health Commission (gzwjkj2022-1-497; gzujkj2017-1-064), the Scientific and Technological in Guiyang City (Grant No. Zhu Ke He [2020]-10-5), and the Public Welfare Technology Research Program in Zhejiang Province (Grant No. LGF21H190001).

## Conflict of interest

The authors declare that the research was conducted in the absence of any commercial or financial relationships that could be construed as a potential conflict of interest.

## Publisher’s note

All claims expressed in this article are solely those of the authors and do not necessarily represent those of their affiliated organizations, or those of the publisher, the editors and the reviewers. Any product that may be evaluated in this article, or claim that may be made by its manufacturer, is not guaranteed or endorsed by the publisher.
