# Supplementary figures and images for: Sensitive and visual identification of Chlamydia trachomatis using multiple cross displacement amplification integrated with a gold nanoparticle-based lateral flow biosensor for point-of-care use
Source: Front Cell Infect Microbiol. 2022 Jul 22;12:949514. doi: 10.3389/fcimb.2022.949514 (PMC9355032; doi:10.3389/fcimb.2022.949514)

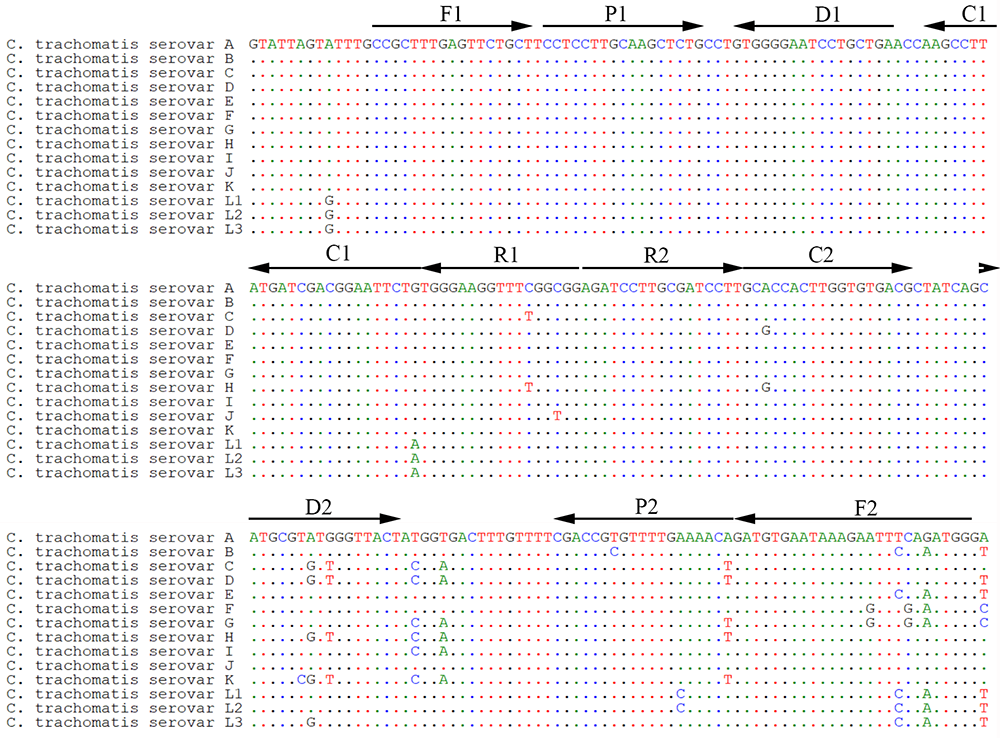

Supplement: Supplementary file 1 [file Image_1.tif]
