# Supplementary material for: Sensitive and visual identification of Chlamydia trachomatis using multiple cross displacement amplification integrated with a gold nanoparticle-based lateral flow biosensor for point-of-care use
Source: Front Cell Infect Microbiol. 2022 Jul 22;12:949514. doi: 10.3389/fcimb.2022.949514 (PMC9355032; doi:10.3389/fcimb.2022.949514)
Supplement: Supplementary file 2 [file Table_1.doc]

**Supplementary Materials**

**FIGURE S1 Nucleotide sequences and location of the *ompA* gene used to design the *C. trachomatis-*MCDA primers**

The nucleotide sequences of the *ompA* gene from 14 *C. trachomatis* serological variants (serovar A, B, C, D, E, F, G, H, I, J, K, L1, L2, L3) were aligned with MEGA-X software, and the MCDA primer sequences are marked with arrows. The right and left arrows showed sense and complementary sequences, respectively.

**TABLE S1:** Comparison of MCDA-AuNPs-LFB and qPCR methods for assessment of *C. trachomatis* in clinical samples

| **Sample NO.** | **qPCR^d^ results（copies）** | | **MCDA-AuNPs-LFB results** |
| --- | --- | --- | --- |
| Test 1 | 4.68×10^5^ | | + |
| Test 2 | 2.21×10^3^ | | + |
| Test 3 | 9.21×10^5^ | | + |
| Test 4 | 615 | | + |
| Test 5 | 4.32×10^4^ | | + |
| Test 6 | 4.56×10^3^ | | + |
| Test 7 | 2.30×10^4^ | | **+** |
| Test 8 | 2.82×10^6^ | | + |
| Test 9 | 4.14×10^5^ | | + |
| Test 10 | 1.69×10^4^ | | + |
| Test 11 | 1.74×10^4^ | | + |
| **Test 12^a^** | **— (~200)** | | **+** |
| Test 13 | 4.75×10^3^ | | + |
| Test 14 | 532 | | + |
| Test 15 | 1.87×10^3^ | | + |
| Test 16 | 1.04×10^3^ | | + |
| Test 17 | 5.82×10^3^ | | + |
| Test 18 | 1.89×10^3^ | | + |
| Test 19 | 646 | | + |
| Test 20 | 2.45×10^3^ | | + |
| Test 21 | 6.64×10^4^ | | + |
| Test 22 | 7.54×10^4^ | | + |
| Test 23  Test 24  Test 25  Test 26  Test 27  Test 28  Test 29  Test 30  Test 31  Test 32  Test 33  Test 34  Test 35  Test 36  Test 37  Test 38  **Test 39^b^**  Test 40  Test 41  Test 42  Test 43  Test 44  Test 45  Test 46  **Test 47^c^**  Test 48  Test 49  Test 50  Test 51  Test 52  Test 53  Test 54  Test 55  Test 56  Test 57  Test 58  Test 59 | 1.96×10^4^  4.23×10^4^  1.17×10^5^  2.78×10^3^  2.57×10^4^  8.64×10^3^  3.47×10^4^  4.21×10^4^  6.69×10^4^  5.97×10^4^  6.59×10^5^  2.68×10^5^  728  3.26×10^3^  3.31×10^3^  3.69×10^5^  **— (~300)**  2.16×10^5^  1.14×10^5^  9.36×10^5^  564  4.37×10^3^  8.85×10^4^  2.17×10^5^  **— (~200)**  4.92×10^6^  6.72×10^4^  1.21×10^5^  1.66×10^3^  1.34×10^4^  7.13×10^5^  1.24×10^6^  2.26×10^6^  2.67×10^4^  3.54×10^7^  769  1.12×10^5^ | | **+**  +  +  +  +  +  **+**  +  +  +  +  +  +  +  +  +  **+**  +  +  +  +  +  +  +  **+**  +  +  +  +  +  +  +  +  +  +  +  + |
| Test 60-135 | | — | — |

Notice: ^a^Test 12, ^b^Test 39, ^c^Test 47: the three samples were amplified with PCR, and the amplicons were tested using DNA sequencing. The results were consistent with MCDA-AuNPs-LFB and presented positive outcomes.

^d^qPCR, the qPCR diagnosis was carried out using commercial real-time TaqMan PCR Kit (DaAn Gene Co., Ltd. China). The concentrations of *C. trachomatis* less than 500 copies will be regarded as negative outcome according to the manufacturer’s instructions.

+, Positive; —, Negative
